# Impact of family-centered care in families with children with intellectual disability: A systematic review

**DOI:** 10.1016/j.heliyon.2024.e28241

**Published:** 2024-03-15

**Authors:** Teresa Dionísio Mestre, Manuel José Lopes, David Matias Mestre, Rogério Ferrinho Ferreira, Ana Pedro Costa, Ermelinda Valente Caldeira

**Affiliations:** a Comprehensive Health Research Centre [CHRC], Portugal; b Polytechnic Institute of Beja – Health Department, Portugal; c University of Évora – Health Department, Portugal; d Local Health Unit of Lower Alentejo [ULSBA], Beja, Portugal

**Keywords:** Disabled children, Family, Family nursing, Health personnel, Intellectual disability, Systematic review

## Abstract

**Background:**

Family-Centered Care (FCC) is an approach to healthcare planning, delivery and evaluation, based on beneficial partnerships between health professionals, patients and families. FCC may be particularly relevant for families with children with intellectual disability (ID), given their needs of continuum care.

**Objective:**

To identify which components of the FCC are practiced and which health outcomes are considered effective in families with children with ID.

**Method:**

A systematic review guided by the PRISMA STATEMENT 2020 approach and the STROBE reporting guidelines was performed on specific databases through the EBSCOhost Web platform: MEDLINE *with Full Text*, CINAHL PLUS *with Full Text*, Academic Search Complete and Psychology and Behavioral Sciences Collection. Peer-reviewed articles published in English or Portuguese languages from 2018 to September 2023 were retrieved. Methodological quality was established using the Quality Assessment Tool for Observational, Cohort and Cross-Sectional Studies – NHLBI, NIH.

**Results:**

Ten studies met the eligibility criteria and were synthetized. The results revealed nine components, reflecting the way FCC was developed: shared decision-making; family education; respect for culture; family engagement; recognition of the family's needs, characteristics and interests; specialized care support; social and emotional support; family functionality; and family seen as a unit. The health outcomes demonstrate effective gains in improving children's health through family satisfaction with health services. Also achieved psychological and social benefits, with improved family well-being and quality of life, favoring family empowerment.

**Conclusions:**

The evidence suggests that FCC components involves an effective partnership between the family and health professionals as the main key in developing care plans, as well as the experience that the family unit brings to the delivery of care. FCC approach include all family members as decision-makers, providing emotional, physical and instrumental levels of support. Health outcomes emerged in three strands; for children with ID, families and health services.

## 1 Introduction

Having a child with an identified intellectual disability or other health condition is a life event that can and often do have negative effects on family's psychological health and well-being [1]. Research indicates that families of children with intellectual and developmental disabilities often experience increased rates of stress (e.g., high medical costs) [2], anxiety [3], depression [4,5] and attenuated psychological well-being [1] in the absence of effective handling mechanisms and social support [[6], [7], [8]]. In addition to these adverse effects on families, raising a child with an intellectual disability (ID) can also have negative effects on the beliefs about their child-rearing confidence and competence [9].

Since all children are predisposed to establishing relationships with their primary carers – usually their parents, who provide physical protection and comfort, the concept of affection becomes vital in this context. The development of affection relationships is recognized as a complex and interactive process between the individual and their primary caregivers, and thought this the sensitivity of parents strongly influences the quality of this relationship [10]. There is evidence that children and young people with ID may be more likely to develop affection difficulties [11]. In this context, parents/family may find it more difficult to identify and meet the needs of ID children [12], or be more stressed, which leads to a decrease in parental involvement. They are also more likely to have mental health problems [11], and the level of interaction and enjoyment they have with their children may be impaired. Such problems can be a general risk factor for developing affection difficulties [10]. In this regard, it is essential to approach affection difficulties through a comprehensive and supportive approach that considers the unique needs of family and children. Professional collaboration and a holistic approach that addresses the emotional, social, and practical aspects of parenting children with ID are key components of effective intervention [10,11].

The level of support and healthcare that a child with ID needs differs in part from the nature and severity of the ID [5]. The disorders of intellectual development are a group of etiologically diverse conditions originated during the developmental period, characterized by significantly low average intellectual and adaptive functioning [13], with an estimated IQ below 70 [14]. It is found that deficits in intellectual and adaptive functioning are the main characteristics of ID with a reported prevalence between 1% and 3% of the population per country [12,13]. Commonly, these people have profound neuromotor dysfunctions, often accompanied by sensory impairments and health problems [[15], [16], [17]]. During childhood, gross motor delay is the most common symptom. In the pre-school and first cycle period, language, learning and difficulty in studies are the most common presentations in children with ID [18].

The widely accepted systems for defining and classifying ID consider adaptive functioning in terms of conceptual, social, and practical domains [13,19]. According to the Diagnostic and Statistical Manual of Mental Disorders (DSM-V), deficits in intellectual function includes reasoning, problem solving, planning, abstract thinking, judgment, academic learning and learning from experience [19]. Inherent to these deficits, the critical components are verbal comprehension, working memory, perceptual reasoning, quantitative reasoning, abstract thinking, and cognitive efficacy [19]. The above deficits in adaptive functioning result in the inability to meet developmental and sociocultural norms for personal independence and social responsibility. Thus, ID may impair adaptive functioning in one or more activities of the daily living, such as communication, social activities, and self-care [[15], [16],^19^] in several settings, as home, school or community [20].

Children with ID are therefore always dependent on others [21]. This dependency means that mostly families/parents play a large, often lifelong [14,22,23] role in the lives of their children. According to these children permanent needs, families/parents experience more distress and require more support than other parents [22]. The impact can be seen in many areas of parent life that it drives them to seek support systems, both within and outside the family. Medical routines, constant vigilance and frequent medical appointments place significant time demands on these families [24]. On-going sleep disturbance is also a common problem for these parents and has been found to be associated with poor mental health and affection difficulties [10], once the burden of care experienced by families can be substantial. Navigating the healthcare system is a challenge for families and can result in a busy appointment schedule with problems of care coordination [24].

Furthermore, the needs of these families seem to be dependent on the characteristics of the parents or caregivers and, most importantly, the children with disability [25]. It is also noted that individual members of a family are so interrelated that any experience affecting one member will affect all [25]. Therefore, families play an integral role providing care to children with health conditions, being imperative to increase this recognition. In this regard, the Social Baseline Theory suggests that the existence of social support is a fundamental aspect of human evolution and well-being [26]. Applying Social Baseline Theory to families with children with ID involves recognizing the importance of social connections and supportive relationships in their lives [27].

Since children with ID require healthcare and supports beyond the ones provided to typically developing children [5], early professional intervention becomes essential [1] and health professionals are therefore recognized both as a powerful source of information and as promoters of skills in these families [28]. By fostering inclusive environments and providing opportunities for positive social interactions, health professionals can contribute to the well-being and development of these children [27].

Some significant theoretical frameworks applied to children with ID focus on social barriers and inclusive environments, by modifying educational and social settings to accommodate the diverse needs of these children and advocating social inclusion [29].The Social Model of Disability has been effective in promoting a more inclusive and equitable approach to disability, however, it has been criticized for placing too much emphasis on physical accessibility, overlooking other aspects of disability, such as social attitudes, stigma, and economic disparities [30]. Similarly, the Ecological Systems Theory emphasizes the interconnection between individuals with ID and their environments through different systems [31]. This theory offers a holistic perspective by considering multiple levels of influence on an individual's development but may not adequately address cultural variations and the unique ways in which cultural factors impact families with children with ID through different ecological systems. Also, may not provide a detailed understanding of how contextual changes, such as changes in social attitudes or economic factors, impact development of children with ID [31,32].

At this level, Family-Centered Care (FCC) emerges as the dominant theoretical framework for healthcare delivery in the pediatric context [1]. It is an approach to the planning, delivery and evaluation of healthcare that is based on beneficial partnerships between health professionals, patients, and families. Requires that the needs of all family members should be identified, addressed, and balanced [25]. The way health professionals interact, care and support families and their children can influence parental self-efficacy and beliefs. Research indicates that the use of the FCC approach by health professionals is positively related to self-efficacy beliefs and families’ feelings of competence and confidence [33]. In their practice, health professionals who employ FCC incorporate five fundamental principles: (1) share information, so families can make informed decisions; (2) develop a constructive working relationship with family members that includes respect for cultural values and practices related to care; (3) engage family members in obtaining resources and support; (4) negotiate and change care plans established with families; and (5) give importance to the family, the school, and quality of life context of patients and their families [28,[34], [35], [36]].

In the meantime, FCC is considered the standard of pediatric healthcare by many clinical practices, hospitals, and healthcare groups [28]. However, based on existing definitions, many FCC models have been proposed for a wide variety of pediatric patient populations. To provide comprehensive healthcare in global pediatric care, the partnership with family members is valued, considering parents as experts when it comes to their children's abilities and needs [37]. In the context of newborn intensive care unit, FCC interventions can facilitate the empowerment of parents in daily care and during a crisis [38]. In the context of stroke, the FCC approach to rehabilitation revealed an improvement in the depression and health status of caregivers of teenagers one year after stroke [39]. Other researchers have argued that the FCC offers an opportunity to support families and strengthen a working partnership between younger children, family, and health professionals in palliative medicine [40]. With an extensive pediatric background and growing number of children living with chronic illness, FCC can help healthcare systems to provide support and improve quality-of-life, for patients and their families.

The benefits can extend to various aspects of the healthcare system and contribute to overall cost-effectiveness [37,41,42], as reduced hospitalization costs (cost savings for the healthcare system emphasizing preventive care and early intervention) [41,43]; efficient resource allocation [41]; increased productivity and employment when families receive adequate support and resources [37]. It also involves education and skill development empowering family's education and skills development to manage their child's condition effectively [37,41]; and promotion of community-based care [42]. Community-based care is commonly more cost-effective than hospital-based care and can contribute to a more efficient use of healthcare resources [42]. From this perspective, FCC seems to be the most effective theoretical approach for families raising children with ID.

However, there is a lack of synthetized evidence specifically on the effect of FCC on families with children with ID. This gap will prevent the understanding and management of the health needs of these families, as well as the development of strategies that allow the family system to function as a unit while responding to the individual needs of its members. Therefore, the objective of this paper was to conduct a systematic review based on the impact and outcomes of the FCC approach in families with children with ID, since it is intended to identify which components of the FCC are practiced and which health outcomes are considered effective in families with these specific characteristics. The defined research questions were the following: What impact does the FCC have on families with children with ID? Which components of the FCC are commonly practiced on families with children with ID? And what are the health outcomes effective in these families?

## 2 Methods

The review protocol of this study was prospectively registered in the International Prospective Register of Systematic Reviews (PROSPERO): CRD 42023398902. This review was reported in accordance with the Preferred Reporting Items for Systematic Reviews and Meta-Analyses (PRISMA STATEMENT 2020) recommendation [44], and the Reporting Guidelines - Strengthening the Reporting of Observational Studies in Epidemiology (STROBE) [45] for cross-sectional studies and cohort studies.

### 2.1 Eligibility criteria

The inclusion criteria were as follows: 1) Population in study, families (parents or other members) with children (aged ≤18 years) diagnosed with ID. All disturbances or disabilities that imply a delay in physical and/or cognitive development were considered, based on the expected for a given age or developmental phase. These perturbations are temporally indefinite and constitute a source of substantial disability, involving biological and non-biological etiology. 2) Parents or other family members should be functional adults with cognitive capacity to participate in the study. 3) Human studies in which the FCC was the approach studied, related to health care needs of families with children with ID. 4) Possibility of including studies comparing the FCC approach/model with its absence, in terms of results. 5) Full-text studies with available references published in English or Portuguese, between January 2018 and September 2023.6) Study methodology and design, quantitative methodologies, considering interventional and observational studies.

The exclusion criteria were: 1) Studies that were abstract-only articles, books, thesis, conference paper, editorial comments, protocols, and review articles. 2) Studies without relation to the theme under study and with ambiguous methodology. 3) Studies with publication date prior to 2018 and repeated in all databases.

### 2.2 Search strategy

Before starting this review, the research was conducted on the PROSPERO and Cochrane platforms to ensure that no other review with the same objectives has ever been published or registered in the planning and execution phase.

After the formulation of the research questions followed the collection of data on the topic under study, which took place between the months of August and September 2023, the authors conducted the literature search using the following databases through the EBSCOhost Web platform: MEDLINE *with Full Text*, CINAHL PLUS *with Full Text*, Academic Search Complete and Psychology and Behavioral Sciences Collection Databases. The final search date was the inception of each database to September 01, 2023. The search terms applied in each database were “Intellectual Disability, Developmental Disabilities, Disabled Children, Family Nursing, Family Centered Nursing, Family Centered Care, Family Centered Practice, Family Centered Approach, Family Centered Intervention, Family Centered”. These keywords were combined with the Boolean operators “AND”, “OR” to get more focused and productive results, in the following order: [(Intellectual Disability) OR (Developmental Disabilities) OR (Disabled Children)] AND [(Family Nursing) OR (Family Centered Nursing) OR (Family Centered Care) OR (Family Centered Practice) OR (Family Centered Approach) OR (Family Centered Intervention) OR (Family Centered)]. Details of the database search strategy deployed in this study are presented on Appendix A.

### 2.3 Study selection

**Fig. 1:**
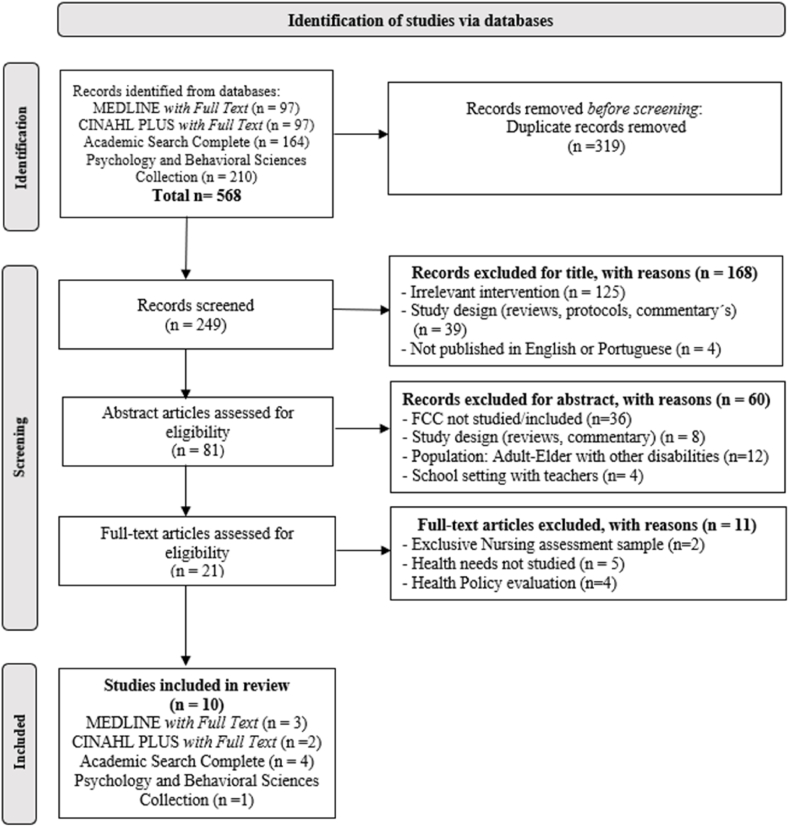
PRISMA flow diagram for study selection.

All retrieved studies were imported into Mendeley Reference Manager to exclude duplicate studies. Next, two reviewers (TDM and DMM) assessed independently the remaining studies titles and abstracts to access their eligibility. A third reviewer (EVC) was invited if there was a difference in opinion between the two reviewers. The retrieved studies were cross checked by the authors TDM and DMM. Finally, the full text was screened and evaluated for eligibility. One reviewer (TDM) extracted data from the included studies and discussed it with a second reviewer (EVC) if further clarification was needed. Each disagreement was resolved through discussion and debate between the reviewers, until a consensus was reached on the inclusion of all the studies. The final inclusion of studies into the systematic review was by agreement of all the authors. The research flowchart based on the PRISMA STATEMENT guidelines (Fig. 1), summarizes the systematic review process.

The research identified 568 studies. After removing the duplicates, 249 were considered eligible for review and screened according to titles. The main reason for excluding these records was the irrelevant reported intervention (n = 125). Plus, the design of the studies did not fulfil the inclusion criteria. Were excluded reviews, protocols, and editorial comments (n = 39), and studies published in other languages than Portuguese or English (n = 4). Then, the evaluation of the eligible articles was carried out in two phases. Firstly, 81 articles were selected after reading the titles, and then the abstracts reading, resulting in 21 articles.

After selecting 21 articles, a full-text reading was made, and 11 studies were excluded. On the inclusion phase, ten articles were selected to the review (Fig. 1), and due to the design of the studies, they were screened according to the Strengthening the Reporting of Observational Studies in Epidemiology (STROBE) Reporting Guidelines [45] for cross-sectional and cohort studies. The reviewers who selected the studies, screened the studies separately, and held a final meeting to reach consensus. Each study was assessed based on its title, abstract, introduction, methods, results, discussion, and other relevant information, such as the perception of the family and health professionals regarding the FCC (Table 1). The main purpose of this analysis was to evaluate the strengths and weaknesses of the included studies.

**Table 1:** STROBE Reporting Guidelines of included studies.

		Bosak et al., 2019 [46]	Cordeiro et al., 2018 [47]	Dias & Cadime, 2019 [48]	Donley et al., 2018 [49]	Gur & Hindi, 2022 [50]	Lucyshyn et al., 2018 [51]	McConkey et al., 2023 [52]	Ogourtsova et al., 2021 [53]	Russel et al., 2018 [54]	Shevell et al., 2018 [55]	Not reported N (%)	Reported N (%)
	**Title and abstract**												
1a	Indicate the study's design in the title/abstract			+					+		+	7 (70)	3 (30)
1b	Provide the abstract an informative and balanced summary of what was done and what was found	+	+	+		+	+	+	+	+	+	1 (10)	9 (90)
	**Introduction**												
2	Explain the scientific background and rationale for the investigation being reported	+	+	+	+	+	+	+	+	+	+	0	10 (100)
3	State specific objectives, including any prespecified hypothesis	+	+	+	+	+	+	+	+	+		1 (10)	9 (90)
	**Methods**												
4	Present key elements of study design early in the paper	+			+	+	+	+	+		+	3 (30)	7 (70)
5	Describe the setting, locations, and relevant dates, including periods of recruitment, exposure, follow-up, and data collection	+			+		+	+		+	+	4 (40)	6 (60)
6	(a)Give the eligibility criteria, and the sources of methods of selection of participants	+		+	+		+		+	+	+	3 (30)	7 (70)
7	Clearly define all outcomes, exposures, predictors, potential confounders, and effect modifiers. Give diagnostic criteria, if applicable	+	+	+	+		+	+	+	+	+	1 (10)	9 (90)
8	For each variable of interest, give sources of data and details of methods of assessment (measurement). Describe comparability of assessment methods if there is more than one group	+		+			+	+		+	+	4 (40)	6 (60)
9	Describe any efforts to address potential sources of bias	+					+			+		7 (70)	3 (30)
10	Explain how the study size was arrived at	+	+	+			+	+	+	+	+	2 (20)	8 (80)
11	Explain how quantitative variables were handled in the analyses. If applicable, describe which groupings were chosen and why	+	+	+	+	+	+		+	+	+	1 (10)	9 (90)
12a	Describe all statistical methods, including those used to control for confounding	+	+	+	+	+	+		+	+	+	1 (10)	9 (90)
12b	Describe ant methods used to examine subgroups and interactions									+	+	8 (80)	2 (20)
12c	Explain how missing data were addressed						+					9 (90)	1 (10)
12d	If, applicable, describe analytical methods taking account sampling strategy	+	+	+			+	+		+		4 (40)	6 (60)
12e	Describe any sensitive analyses	+										9 (90)	1 (10)
	**Results**												
13a	Report numbers of individuals at each stage of study-eg, numbers potentially eligible, examined for eligibility, confirmed eligible, included in the study, completing follow-up, and analyzed	+		+	+		+	+	+	+	+	2 (20)	8 (80)
13b	Give reasons for non-participation at each stage	+					+	+			+	6 (60)	4 (40)
13c	Considerer use of a flow diagram	+				+						8 (80)	2 (20)
14a	Give characteristics of study participants (eg, demographic, clinical, social) and information on exposures and potential confounders	+	+	+	+	+		+	+	+	+	1 (10)	9 (90)
14b	Indicate number of participants with missing data for each variable of interest										+	9 (90)	1 (10)
14c	To cohort-study – Summarize follow-up time (eg, average and total amount)						+					–	1 (10)
15	Report numbers of outcome events or summary measures	+	+	+	+	+	+	+	+	+	+	0	10 (100)
16a	Give unadjusted estimates and, if applicable, confounder-adjusted estimates and their precision (eg, 95% confidence interval) Make clear which confounders were adjusted for and why they were included		+		+		+			+	+	5 (50)	5 (50)
16b	Report category boundaries when continuous variables were categorized									+		9 (90)	1 (10)
16c	If relevant, consider translating estimates of relative risk into absolute risk for a meaningful time period		+									9 (90)	1 (10)
17	Report other analyses done-eg analyses of subgroups and interactions, and sensitivity analyses			+						+	+	7 (70)	3 (30)
	**Discussion**												
18	Summarize key results with reference to study objectives	+	+	+	+	+	+	+	+	+	+	0	10 (100)
19	Discuss limitations of the study, taking into account sources of potential bias or imprecision. Discuss both direction and magnitude of any potential bias	+	+		+	+	+		+	+		3 (30)	7 (70)
20	Give a cautious overall interpretation of results considering objectives, limitations, multiplicity of analyses, results from similar studies, and other relevant evidence	+		+	+	+	+	+		+	+	2 (20)	8 (80)
21	Discuss the generalizability (external validity) of the study results	+				+	+		+	+	+	4 (40)	6 (60)
	**Other information**												
22	Give the source of funding and the role of the funders for the present study and, if applicable, for the original study on which the present article is based		+	+	+		+	+	+	+		3 (30)	7 (70)
	**Number of items**	**23**	**15**	**18**	**16**	**13**	**24**	**16**	**17**	**24**	**22**		

### 2.4 Data extraction

Data were extracted with a report table, including: 1) authors, year of publication and country; 2) study design; 3) study aims; 4) study setting; 5) sample; 6) approach or form of evaluation of the FCC; 7) the FCC components; 8) the identified FCC outcomes; 9) the main key findings; and 10) the studies quality rating (Table 2). Two reviewers (TDM and APC) performed data extraction and data synthesis independently. If there were disagreements, the results were determined by the third reviewer (EVC) after discussion.

**Table 2:** Summary of included studies in the Review.

Authors/Year/Country	Design	Aim of the study	Setting	Sample	FCC evaluation	FCC components	Effective FCC Outcomes	Key Findings	Quality Rating
[46] Bosak, Jarvis & Khetani/2019/Chicago - USA	Descriptive Study	Report the proportion of caregivers that created multiple care plans using PEM+, an electronic health tool.	Home and Community participation	N = 18 parents/caregivers	FCC approach supported through caregiver capability to design an initial plan of care using PEM+	Parental engagement in care process Shared decision making Caregiver education	- Improved child outcomes: in rehabilitation -Satisfaction with health services -Improved accessibility to healthcare	-Most caregivers created multiple care plans using PEM+, suggesting caregiver interest in engaging in the process of FCC using an electronic health option. -Developed high-quality care plans	Fair (54.5%)
[47] Cordeiro, Davis, Antonelli, Rosenberg, Kim, Berhane & Turchi/2018/San Francisco - USA	Observational Cross-Sectional Study	(1) identify associations among receipt of adequate care coordination with family-provider relations and child outcomes (2) compare these associations to previous survey findings.	Data from the 2009–2010 National Survey of Children with Special Health Care Needs	N = 400 parents divided on 4 groups	FCC evaluated trough parents' perception in association to care coordination	Family engagement in care process Shared decision making	-Satisfaction with health services -Improved child outcomes -Psychological benefits: decreased parental stress	-Group 2 increased odds of receiving FCC and experiencing partnerships with professionals and satisfaction with services. -Having adequate care coordination continues to be associated with receipt of FCC partnerships with professionals and satisfaction with services.	Fair (54.5%)
[48] Dias & Cadime/2019/Portugal	Descriptive study	Explore families and practitioners' perspectives about child/family centered practices and related variables	Community participation (Schools and Health Centers)	N = 78 families N = 60 practitioners of early intervention	FCC evaluated through families' perceptions. Was applied the Portuguese adaptation of the Family Focused Intervention Scale	Family engagement in care process Family education Specialized childcare support	-Satisfaction with health services -Social family benefits -Improved family well-being and quality of life	-Practitioners most frequent practices were centered on providing information and instructional activities. -Practices were more centered on the children than centered on families. -Families reinforced that centered practices were the most prevalent.	Fair (50%)
[49] Donley, King, Nyathi, Okafor & Mbizo/2018/Florida - USA	Observational Cross-Sectional Study	Demonstrate how children mental health and developmental needs affect parent parenting, adult mental health, and family relationships, reducing their capacity for protection and resilience	Data from the 2011–2012 National Survey of Children's Health	N = 5503 parents	FCC Model for delayed care in children with special health needs	Specialized childcare support Social and emotional support Family functionality Respect for cultural values	-Delayed healthcare reduced -Improved accessibility to healthcare -Social family benefits	-Family dynamics have a greater impact on delayed care than socioeconomic factors. -The use of qualified mental health professionals described in the proposed FCC model can positively affect family support reducing the presence of care delays.	Poor (41.5%)
[50] Gur & Hindi/2022/Israel	Descriptive study	To study parents' perspectives on FCC/services for families of children with disabilities focusing on their use and experiences with healthcare services.	Community approach through an online survey	N = 33 parents (male)	FCC approach was the theoretical framework evaluated through parents′ perspectives	Family engagement in care process Recognition of the family's needs, characteristics, and interests	-Psychological, familial, and social benefits -Improved family well-being and quality of life -Family empowerment	-Most fathers said the Israeli FCC program fit their needs. -Participation in the program yielded psychological, familial, and social benefits. -Family centered services should make special efforts to reach out to fathers.	Poor (30%)
[51] Lucyshyn, Miller, Cheremshynski, Lohrmann & Zumbo/2018/Canada	Observational Cohort study	Family functioning results from the second half of a longitudinal study that investigated the consequential validity of an ecological approach to family-centered positive behavior.	Home and Community participation	N = 10 families with child with developmental disability	FCC evaluation through family functioning measures: Family Quality of Life Survey; Parental Stress Index; Parental Locus of Control Scale; Social Support Questionnaire	Family functionality Family seen as a unit	-Improved family well-being and quality of life -Psychological benefits: decreased parental stress	-Significant and lasting improvements in the perception of parental stress by fathers and mothers. -Mothers' improvements in satisfaction with the family's quality of life and internal locus of control suggest the value of carrying out an ecological assessment of the family in conjunction with a functional assessment. -Mothers satisfaction with internal locus of control	Fair (71.5%)
[52] McConkey, O'Hagan & Corcoran/2023/Ireland	Descriptive Study* + Qualitative (telephone structured interview)	1)Participation in community activities of children with intellectual disability 2)Recognize emotional support to parents/families 3)Boost the resilience and capacity of parents to cope daily challenges	Home and Community participation	N = 96 families with 110 children	FCC evaluated through parents self-completed rating scales on social participation, and their emotional and social well-being	Family engagement in care process Social and emotional support Parental perceptions in care process	-Psychological benefits: parental improved confidence and resilience -Improved parental well-being -Social family benefits	-Parents reported higher well-being scores and improved social engagements outside of the home (except for the COVID-19 period). - Children developed skills through activities at home and involvement in community activities.	Fair (54.5%)
[53] Ogourtsova, O'Donnell, Chung, Gavin, Bogossian & Majnemer/2021/Canada	Descriptive Study* + Qualitative (semi-structured interview)	For father-participants, the survey aimed to gather information about their involvement and perceptions in the healthcare of their children regarding experiences and interactions with health professionals	Healthcare Hospitals data source	N = 7 parents (male) N = 13 health professionals	FCC seen as facilitator on the interaction of parents and health professionals	Family engagement in care process Family education Respect for cultural values Family seen as a unit	-Psychological and social benefits -Improved accessibility to healthcare	-The fathers reported to be moderate to very much involved in the healthcare of their children. -The suggestions are related to communication strategies, changes in clinical practices using FCC approach and consideration of cultural differences. -Satisfaction and comfort in interactions.	Poor (33.5%)
[54] Russell, Beckmeyer & Su-Russell/2018/USA	Observational Cross-Sectional Study	Through social determinants of health framework understand how family structure can affect parental perceptions of FCC and its associations with positive developmental outcomes for young people with special healthcare needs.	Data from the 2011–2012 National Survey of Children's Health	N = 8740 parents	FCC perceptions were associated with three positive developmental outcomes among youth with special health care needs.	Family engagement in care process Parental education Parental perceptions in care process	-Improved child and youth outcomes -Family empowerment	-Married biological parents perceived greater FCC than parents in other family structures. -The association between perceptions of FCC and youth developmental outcomes were strongest in married biological families. -Family nurses essential for health outcomes. -Families reduced perception of FCC.	Good (82%)
[55] Shevell, Oskoui, Wood, Kirton, Rendburg, Buckley, Ng & Majnemer/2018/Canada	Observational Cross-Sectional Study	To identify characteristics of children with cerebral palsy, intrinsic and extrinsic factors that may be associated with parents' perceptions of FCC health services.	Data from Canadian Cerebral Palsy Registry	N = 282 parents	Focus on child and environmental factors that influence the extend of FCC provided by heath teams	Family engagement in care process Social and emotional support Recognition of the family's needs, characteristics, and interests Family seen as a unit Parental perceptions in care process	-Satisfaction with health services -Improved child outcomes: development and psychological adjustment -Psychological benefits	-Sociodemographic factors were associated with parental perceptions of FCC. -Factors intrinsic to the child's cerebral palsy were not associated with parental perceptions.	Fair (58.5%)

*Only data from the descriptive study will be used in the present Systematic Review.

### 2.5 Quality assessment of included studies

The two reviewers TDM and APC assessed the included studies′ quality independently and, if there was divergence, a third reviewer (RFF) was invited.

The quality analysis of the studies was according to the National Institutes of Health (NIH) Quality Assessment of Controlled Intervention Studies, which follows a 14-item checklist [56]. The checklist was designed to help reviewers focus on the key concepts for evaluating the internal validity of a study. The critical appraisal involves considering the risk of potential for selection bias, information bias, measurement bias, or confounding (the mixture of exposures that one cannot tease out from each other). High risk of bias translates to a rating of poor quality. Low risk of bias translates to a rating of good quality (thus, the greater the risk of bias, the lower the quality rating of the study) [56]. The quality rating was classified into the three available categories: Poor <50%, Fair 50–75%, and Good ≤75%. The maximum quality rating achieved in this assessment was 82% (Good Quality) and the minimum was 30% (Poor Quality) (Table 3). The quality assessment of the studies had an average score of 0.529 (quality rating about 53%). Six studies were classified as fair [[46], [47], [48], [51], [52], [55]], three as poor [[49], [50], [53]], and one as good [54] elucidating on the support in scientific evidence and the foundations for ongoing research. All of them clearly stated the research question or objective and the outcome measures across the participants. Nine studies described the characteristics of the participants (e.g., demographic, and social) and provided information on exposures and potential confounders [[46], [47], [48],[49], [50], [52], [53], [54], [55]]. The study by Lucyshyn et al. [51] was the only one that explained how missing data were treated. And only the study by Cordeiro et al. [47] considered transforming relative risk estimates into absolute risk during a significant study period (Table 3).

**Table 3:** Quality assessment tool for observational, cohort and cross-sectional studies.

Year	Author	1	2	3	4	5	6	7	8	9	10	11	12	13	14	Total Score	Quality Rating
2019	Bosak et al. [46]	Y	Y	NR	Y	Y	NA	NA	N	Y	N	Y	N	NA	N	6/11(54.5%)	Fair
2018	Cordeiro et al. [47]	Y	Y	Y	NR	Y	N	N	NA	N	N	Y	NA	NA	Y	6/11 (54.5%)	Fair
2019	Dias & Cadime [48]	Y	Y	CD	Y	N	NA	NA	NA	Y	N	Y	N	NA	N	5/10 (50%)	Fair
2018	Donley et al. [49]	Y	Y	NR	Y	Y	N	N	NA	N	N	Y	NR	NA	N	5/12 (41.5%)	Poor
2022	Gur & Hindi [50]	Y	N	NR	Y	N	NA	NA	NA	N	N	Y	N	NA	N	3/10 (30%)	Poor
2018	Lucyshyn et al. [51]	Y	Y	NR	Y	N	Y	Y	Y	Y	Y	Y	N	Y	N	10/14 (71.5%)	Fair
2023	McConkey et al. [52]	Y	Y	Y	Y	N	NA	NA	NA	N	Y	Y	N	Y	N	6/11 (54.5%)	Fair
2021	Ogourtsova et al. [53]	Y	N	N	N	Y	NA	NA	NA	N	N	Y	NA	NA	N	3/9 (33.5%)	Poor
2018	Russell et al. [54]	Y	Y	Y	Y	Y	Y	N	NA	Y	N	Y	NA	NA	Y	9/11 (82%)	Good
2018	Shevell et al. [55]	Y	Y	Y	Y	Y	N	N	NA	Y	N	Y	N	NA	N	7/12 (58.5%)	Fair

**Quality of included studies was assessed using the National Institutes of Health (NIH) Quality Assessment tool for Observational Cohort and Cross-Sectional Studies** (https://www.nhlbi.nih.gov/health-pro/guidelines/in-develop/cardiovascular-risk-reduction/tools/cohort). **1**. Was the research question or objective in this paper clearly stated? **2**. Was the study population clearly specified and defined? **3**. Was the participation rate of eligible persons at least 50%? **4**. Were all the subjects selected or recruited from the same or similar populations (including the same period)? Were inclusion and exclusion criteria for being in the study prespecified and applied uniformly to all participants? **5**. Was a sample size justification, power description, or variance and effect estimates provided? **6**. For the analyses in this paper, were the exposure(s) of interest measured prior to the outcome(s) being measured? **7**. Was the timeframe sufficient so that one could reasonably expect to see an association between exposure and outcome if it existed? **8**. For exposures that can vary in amount or level, did the study examine different levels of the exposure as related to the outcome (e.g., categories of exposure, or exposure measured as continuous variable)? **9**. Were the exposure measures (independent variables) clearly defined, valid, reliable, and implemented consistently across all study participants? **10**. Was the exposure(s) assessed more than once over time? **11**. Were the outcome measures (dependent variables) clearly defined, valid, reliable, and implemented consistently across all study participants? **12**. Were the outcome assessors blinded to the exposure status of participants? **13**. Was loss to follow-up after baseline 20% or less? **14**. Were key potential confounding variables measured and adjusted statistically for their impact on the relationship between exposure(s) and outcome(s)?.

**Total Score:** Number of yes; **CD,** cannot be determined; **NA,** not applicable; **NR,** not reported; **N,** no; **Y,** yes.

**Quality Rating:** Poor <50%, Fair 50–75%, Good ≥75%.

### 2.6 Data analysis

A narrative synthesis was performed, given the heterogeneous nature of the interventions involved, the instruments for evaluating FCC, and the health outcomes investigated across the selected studies. A descriptive framework was made to organize the narrative synthesis [57]. To characterize this narrative synthesis: was explained how the FCC approach works and for whom; was developed a preliminary synthesis; were explored relationships within and between studies; and assessed the health outcomes [57]. This framework included nine key FCC core components, categorized according to the five fundamental principles of the FCC, mentioned in the introduction. It served as a guide for presenting the synthesis in a structured manner. The health outcomes were based on the findings highlighted in the studies, which were also attached in the FCC core components identified for the population under study.

The FCC components were extracted from each study as well as their effectiveness on health outcomes. Due to the complementarity on the identified components, authors have decided to aggregate them into four key elements which represent the development and implementation of the universal FCC model [37]. The key elements comprise collaboration/partnership between families and health professionals; consideration of family context; family support needs; and education of families and health professionals.

Health outcomes were subsequently identified in relation to the FCC components. Since most of the components reflected the way in which health professionals and families established a relationship of mutual trust and respect, the outcomes emerged in three ways: in the children, in families and in health services.

## 3 Results

### 3.1 Study and subject characteristics

Regarding the included studies, four were carried out in the United States of America (USA) [46,47,49,54]; three in Canada [51,55,53]; two in Europe, more precisely Ireland [52] and Portugal [48]); and one in Israel [50]. The publications are comprised between the years of 2018 and 2023, being mainly of the year 2018. Five are observational and five are descriptive studies – four are observational cross-sectional [47,[49], [52], [55]], and one refers to a observational cohort study [51] correctly identified in the methodological stage; two descriptive studies have a mixed methodology - quantitative and qualitative [52,53], in which only their descriptive components were considered and analyzed, and the other three descriptive studies only describe the characteristics of the population or phenomenon studied [46,48,50].

All the studies refer directly to the FCC, as an intervention or approach perceived by caregivers and health professionals in families with children with ID.

The main objective in five studies was to highlight the perceptions and perspectives of parents/caregivers on FCC [50], focused on experiences and access to healthcare services [48,50] and/or coordination of care [47], more specifically with health professionals involved in the care of their children with effective diagnosis [47,48,55,50,53]. Four of them aimed to assess the FCC by studying the family context: family functioning [51,52,49]; and family structure [54], associated with positive results in the children's development. Only one of the studies aimed to validate the creation of individualized care plans by caregivers, as well as their characteristics, using an electronic health tool in parents targeted by the FCC approach [46] (Table 2).

### 3.2 Sample size

As for the sample size, the studies are divided into parents/caregivers (n = 15 061) and families (n = 184), being impossible to define how many persons were involved in the family studies. Seven studies are with parents or caregivers [46,47,[49], [50], [53], [54], [55]] and the three others with families [[48], [51], [52]]. Looking separately, two studies [49,54] can be classified as average (more than 1000 but less than 25 000 participants), and the remaining five as small (less than 1000 participants) where the variation is between N = 7 and N = 400 [47,53]. The study by Ogourtsova et al. [53] distinguishes two distinct samples: seven parents mentioned above and thirteen health professionals (Table 2).

If we look at the age of children with ID, all the studies mention that they are between zero and seventeen years old. In four studies the pediatric age was accepted in its entirety [47,[49], [50], [53]]. One study focused adolescent aged twelve to seventeen years old [54], two with children between three and eight years old [48,51], two specifically with children between zero and five years old [46,55] and one between nine months and thirteen years old [52]. In addition to the fact that all the studies concerned families with children with ID, three encompassed the concept of children with special health care needs [47,49,54], and one refers specifically to children with cerebral palsy [55].

The data was obtained from two different settings. Eight studies through community participation [[46], [47], [48], [51], [52],^49^,^50^,^54^] and two through institutional data: Canadian Hospitals [53] and the Canadian Cerebral Palsy Registry [55] (Table 2).

### 3.3 Data collection instruments

The main data collection instruments were validated scales with known reliability and validity. But data, were also achieved from national child health surveys (with special health care needs) in the USA [47,49,54]. One study applied an electronic tool that supports FCC - Participation and Environment Measure Plus (PEM+) [46], by giving caregivers a way to help plan their child's care online, and the others applied Portuguese adaptation of the Family Focused Intervention Scale [48]; Sociodemographic questionnaires [48,52,50,53]; the MPOC-56 questionnaire to mothers and fathers, using data from the Cerebral Palsy Registry [55]; the TWarwick-Edinburgh Mental Well-being Scale [52]; the Family Quality of Life Survey [51]; the Parenting Stress Index [51]; the Parental Locus of Control Scale [51]; and the Social Support Questionnaire [51], which sought to validate the FCC approach.

Through these instruments, two studies made associations between family's characteristics and coordination of care [47], or accessibility to healthcare services [49]. Associations were also made between parents' perception of the FCC delivered and the positive results in their children's development [48,54]. The MPOC-56 questionnaire, through measures of the care process, assessed perceptions of the care the children received, specifically the FCC associated with the behaviors of health professionals [55]. The studies of Ogourtsova et al. [53] and Gur & Hindi [50] intended to analyze the involvement of fathers in the healthcare of their children and the degree of satisfaction with the health professionals involved. Only one study used the electronic health tool Participation and Environment Measure Plus (PEM+) to support the FCC, giving parents digital guidance in planning care for their children [46]. All studies reported the psychometric characteristics of the data collection instruments and/or cited their validation studies.

### 3.4 FCC- core components

The extracted data was analyzed to identify the components of the FCC approach in families with children with ID. Nine components were identified, reflecting the way FCC was developed and implemented in the studies: shared decision-making (n = 2 studies) [46,47]; caregiver/family education (n = 4 studies) [46,48,53,54]; respect for cultural values (n = 2 studies) [49,53]; family engagement in the care process (n = 8 studies) [[46], [47], [48],^52^,^55^,[50], [53], [54]]; recognition of the family's needs, characteristics and interests (n = 3 studies) [52,55,50]; specialized childcare support (n = 2 studies) [48,49]; social and emotional support (n = 5 studies) [[48], [49], [51], [52], [55]]; family functionality (n = 2 studies) [51,49]; and family as a unit (n = 3 studies) [51,55,53] (Table 2). These components validate the perception and perspective of caregivers/families and health professionals, reflecting their reciprocity in the healthcare provided.

#### 3.4.1 Sharing information for family decisions

##### 3.4.1.1 Shared decision-making

The shared decision-making was identified in two studies [46,47] being implicit in the sharing of information between those involved in the care process. It involves encouraging and expressing the preferences and values of parents/caregivers [46,47], through appropriate coordination of care [47], in which both sides (professionals and families) share responsibility for deciding on the best care option to achieve positive health outcomes in children [46].

#### 3.4.2 Development of constructive working relationships with family

##### 3.4.2.1 Caregiver/family education

The importance of the caregiver/family education in the implementation of FCC was identified in four studies [46,48,53,54]. Education was approached from the concept of mutual learning [46,53], in which families and health professionals learn and support each other. Bosak et al. [46] suggest the use of e-health tools by caregivers as an effective form of communication between stakeholders. The knowledge acquired by caregivers translates into positive results for their children's development [54], reflected in practices such as sharing information and focus on health education activities [48].

##### 3.4.2.2 Respect for cultural values

The religious and cultural background of the parents/family influences the provision of care to the child as a component associated with the implementation of FCC [49,53]. Health professionals point out that family-centered practice should allow parents to follow their beliefs, spirituality, and culture when it comes to understand their feelings and needs [49]. Of these cultural factors, traditional beliefs about the father's role in the upbringing and care of the child accounted for 20.7% of all barriers to the implementation of FCC in the study by Ogourtsova et al. [53], predominantly transmitted by health professionals.

#### 3.4.3 Engaging family in obtaining resources and support

##### 3.4.3.1 Family engagement in the care process

The effective partnership of caregivers/family in the care process was the component highlighted in the largest number of studies (n = 8) [[46], [47], [48],^52^,^55^,[50], [53], [54]]. Family engagement in the care process was mentioned as being necessary throughout the care trajectory [46,47,55,54], contributing to the ability of families to maintain control over their child's care plans [46,48,53] and the daily provision of care [48], particularly as care becomes increasingly complex [46]. This collaboration presumes an effort to include fathers in daily care [50] and to develop them in home and community context [52]. The use of e-health tools [46] has highlighted this close partnership.

#### 3.4.4 Negotiation and changes in care plans established with the family

##### 3.4.4.1 Recognition of the family's needs, characteristics, and interests

Recognizing the needs, characteristics and interests of families is another component associated to the FCC approach. This recognition was specifically identified in three studies [52,55,50]. Besides the individual learning targets set for each child (according to their developmental levels), parents and professionals assessed their progress target-by-target [52,55]. However, to obtain health outcomes on the children development, the personal goals of their parents, siblings and other family members were also analyzed [52,50], and an attempt was made to understand the extent to which they could be achieved [52]. Considering the needs and characteristics of families with children with more severe disabilities, it was found that they were more likely to use health services [50], making it imperative to identify what motivated or prevented them from using services [52,50].

##### 3.4.4.2 Specialized childcare support

The specialized childcare support figures in two studies [48,49], associated with early intervention in children with developmental or intellectual disabilities [48] There is a continuous commitment to training health professionals, which requires resources [48], teaching [48] and opportunities that respond to the needs of the population they serve [48]. Evidence shows that the use of health professionals specialized in mental health can positively affect family support, reducing the presence of care delays [49].

#### 3.4.5 Family significance and quality of life

##### 3.4.5.1 Social and emotional support

The support network for the families under study has also been validated, both in social and emotional terms in five studies [[48], [49], [51], [52], [55]]. Families of children with special needs face more financial burdens associated with childcare [49], so social support has an impact on their well-being, confidence, and resilience [48,52]. The study by Shevell et al. [55] explored sociodemographic factors associated with parental perceptions of FCC, associating provision of care in pediatric rehabilitation settings to families with high socioeconomic backgrounds.

The support networks have also shown to mediate the burden experienced by families with special needs [51,49]. Respite care, support for siblings, or psychological counseling are also associated with improvements in overall family functioning [51]. Through this component of FCC, parents' confidence in managing their children has improved; children have developed new skills and have become more connected to community activities [48].

##### 3.4.5.2 Family functionality

Two studies reported a significant impact on health and quality of life of children with ID through family functioning [51,49]. Donley et al. [49] tested the hypothesis that family functioning has a significant effect on delayed healthcare, concluding that its impact is more significant than socioeconomic factors. The fact of being a mother or a father also showed differences in the results of family functioning, since mothers played the role of caregiver earlier and more frequently during the intervention phase of the study [51]. Family functioning outcomes through a family-centered ecological approach showed significant and lasting improvements in parental stress, satisfaction with quality of life and internal locus of control [51].

##### 3.4.5.3 Family as a unit

In the FCC approach, the family seen as a unit is one of the universal principles. This component was identified in three studies [51,55,53]. The family-centered ecological approach emphasizes assessing the overall functioning of the family (with mothers and fathers) to understand the intervention impact on the family as one [51]. If one of the challenges to improving FCC is the manifest uniqueness of families [51,55], adopting this model is essential in pediatric rehabilitation services [55] and in all interactions between health professionals and parents of children with ID [53].

### 3.5 FCC- health outcomes

After identifying the FCC components, it was possible to extract from each study their effectiveness on health outcomes. All the studies reported outcomes inherent to the family-centered approach. Six categories of outcomes were identified in families with children with ID, translated into effective health gains: 1)improved child outcomes; 2) satisfaction with health services; 3)psychological benefits; 4)social benefits; 5) improved family well-being and quality of life; and 6)family empowerment (Table 4).

**Table 4:** FCC health outcomes.

Study FCC health outcomes	Bosak et al., 2019 [46]	Cordeiro et al., 2018 [47]	Dias & Cadime, 2019 [48]	Donley et al., 2018 [49]	Gur & Hindi, 2022 [50]	Lucyshyn et al., 2018 [51]	McConkey et al., 2023 [52]	Ogourtsova et al., 2021 [53]	Russel et al., 2018 [54]	Shevell et al., 2018 [55]
Improved child outcomes	High quality care plans; Rehabilitation	Less school days missed and less domiciliary visits because of illness	–	–	–	Improved child behavior through parent-child interaction	Children involvement in community activities	–	Children health; participation in extracurricular activities and flourishing	Rehabilitation settings in the early phases of care
Satisfaction with health services	Engagement in participation; Focused care planning Improved accessibility to healthcare with an electronic health tool	Partnerships with health professionals; Ease of getting referrals	Frequency of practices focused on personnel family assistance; Positive evaluation of the satisfaction with early childhood intervention services	Improved accessibility to healthcare through coordination of care	–	Satisfaction and comfort level in interactions with HP	–	Family engagement in care process; Respect for cultural values Improved accessibility to healthcare through the father involvement	Improved accessibility to healthcare	Family engagement in the care process; Coordination and comprehensive care for the child and family
Psychological benefits	–	Shared decision making; Decreased parental stress	–	–	Fathers mentally recharge and grow; Positive feelings; Effective family communication; Development of positive coping skills	Decreased parental stress; improved parental locus of control	Parental improved confidence and resilience	Communication strategies; Sharing responsibilities	–	Communication skills; Providing specific information about the children
Social benefits	–	–	Improve resource assistance	Provision of social support and network; Perceived control	Opportunities for shared experiences; Social recognition of fathers	–	Family social inclusion; Home-based support	Social recognition of fathers	–	–
Improved family well-being and quality of life	–	–	Providing information and instructional activities to the family	Family functioning and relationship dynamics has significant impact in quality of life; Sense of stability	Strengthening the family; Family centered approach	Positive behavior support; Family interaction; Emotional well-being; family functioning through FCC ecological approach; Parent-child interaction	Building the competence and resilience of parents; Relationship with health professionals	–	–	–
Family empowerment	–	–	–	–	Family centered approach encourage fathers to use the health services	–	–	Empowering interactions between parents and health professionals; Long lasting relationships	Engagement in care process; Caregivers' knowledge; Mindful parenting	–

One of the most frequently reported outcomes was the child's development, including social [47,52,55], behavioral [51] and rehabilitation [46,55] positive outcomes. Parent/family satisfaction with the health services was other frequently reported outcome, evidenced through partnership and interaction with health professionals in the care process [[46], [47], [48], [51],^55^,^53^], accessibility to healthcare with improvements in parental knowledge and empowerment [46,55,49,53,54]. The psychological and social benefits for families have been achieved through improved communication strategies [55,50,53], sharing of responsibilities [47,50,53], provision of social support networks [48,49] and social inclusion/recognition of the father in the childcare process [52,50,53]. There is evidence that parental stress decreased [51], and family's confidence and resilience increased [52]. The outcome associated with improved family well-being and quality of life was identified in five studies [[48], [51], [52],^49^,^50^], based on family functionality [51,49], relationship dynamics [48,52,49], and family emotional well-being [51,50]. The family empowerment was identified in three studies [[50], [53], [54]], highlighting long-lasting relationships [53], which foster the development of parental knowledge [54] and the father encouragement to use health services [50] (Table 4).

## 4 Discussion

This review investigated the FCC components and their effectiveness in translating health outcomes in families with children with ID. It was possible to identify nine components categorized into the five fundamental principles of FCC.

On the first FCC principle, the shared decision-making to achieve the best care option to children with ID was supported by two studies [46,47]. These findings highlight the shared responsibility established between health professionals and families/caregivers. Especially in the families studied, encouraging and expressing the parents preferences/values were the most important elements to provide adequate shared decisions [46,47]. This approach improves interpersonal relationships between the provider and family, which has led several major organizations, such as the American Academy of Pediatrics, to adopt and promote this model of care [58]. In fact, FCC approach represents a commitment by health professionals to include all family members as decision-makers [58], providing emotional, physical, and instrumental levels of support to the families [37], considering their needs and preferences [25,35].

The second principle evidenced the development of constructive working relationships with the family through caregiver and family education mediated by the concept of mutual learning [46,48,53,54], and by respect for cultural values that allow these families to follow their beliefs, spirituality and culture [49,53]. Mutual learning as a method treats the outcomes and knowledge production processes as interconnected. Only when all participants - families and health professionals - can equally contribute, feel understood, make decisions, and determine priorities together can a transformative « space » emerge. Mutual learning, as a collaborative practice, generates insights and learnings at the dynamic intersection of different ways of being and knowing [59].

The third principle involved engaging family in obtaining resources and support [47,52,55] as a contribute to the ability of families to maintain control over their child's care plans [46,48,[50], [53], [54]]. The engagement between health professionals and families is highlighted as the key factor in developing childcare plans [37], through effective partnerships [47,48,55,53], and based on the development of communication skills [55] and adaptive strategies [53]. Parents have developed high-quality care plans, using an effective electronic health option [46]. This tool supported continuous communication, bringing parents and children closer to health services. Also, through FCC programs most parents have seen their needs [47,52,50,54], and cultural differences [53] addressed. In fact, the FCC approach recognizes the partnership as the main key to developing care plans [37,58], as well as the experience that the family unit brings to the delivery of care [51,55,60,61]. It considers and plans care involving the whole family, rather than the individual child [51,55,53,62].

The fourth supported the negotiation and changes in care plans established with the family through the recognition of these family's needs, characteristics and interests [52,55,50], offering specialized childcare support in response to the needs of children with ID [48,49].

FCC is not just about the physical involvement of families in the care and tasks of the child, it implies a change in culture and relationships, including an individualized approach to supporting and empowering families [50,63]. Psychosocial support resources and interaction with other families with children with ID [[48], [49], [51], [52], [55],^64^], help families to make the best choices and promote attachment strategies with the disabled child [65]. Successful implementation of FCC is more often observed when families interact with other families that have the same case or diagnosis in children [66]. Additionally, working together with specialized health professionals promotes the child inclusion in daily activities, whether at home or in the community [67,68]. To this end, there has been an effort in the USA to collaborate with professional organizations to identify core cross-disciplinary competencies for all personnel working with children with disabilities and their families [69]. Also in Europe, a movement of cooperation between families and health professionals has already been set up [70].

The fifth principle highlighted the quality of life context and significance given to the families with children with ID, through social and emotional support [[48], [49], [51], [52], [55]] with improvements in family functionality [51,49], as a unit [51,55,53]. A wealth of international research shows that developing parents and family's competence and resilience and increasing their personal well-being is crucial to ensure good outcomes for children and their physical, social, cognitive, and emotional growth [65,71,72]. The family's ability to nurture, care for, protect, teach and influence throughout life makes it an effective entry point for maintaining individual and collective health and an important component of community and public health [61,73].

From all the above categorized components and previous research about FCC approach, four key elements emerged to families with children with ID [1,37,41,51,60]. These key elements are a summary of the evidence described in the studies included in the review. Comprises collaboration/partnership between families and health professionals [[46], [47], [48],^52^,^55^,[50], [53], [54]]; consideration of family context [46,49,53]; family support needs [[48], [49], [50], [51], [52], [55]]; and education of the family and health professionals to care children with ID [46,48,49,53,54].

Considering the identified FCC components and the key elements in the specific families studied, the health outcomes emerged in three ways: 1) in the children with ID [46,47,[51], [52], [55],^54^], 2) in families (considered as a unit, with benefits in all members) [[47], [48], [49], [50], [51], [52], [53], [54], [55]] and 3) in health services [19,46,47,51,55,49,53,54]. Specifically for these children, there have been social [47,52,54], behavioral [51] and rehabilitation improvements [46,55]. For the families, the psychological and social benefits were reflected in improved family well-being and quality of life [[48], [51], [52],^49^,^50^], mainly due to the support network provided [[48], [51], [52]]. In health services, the family's perceptions related to the care received: satisfaction with the health services [[46], [47], [48], [51],^55^,^53^] and accessibility [46,55,49,53,54] were emphasized. Regarding accessibility, the PEM + electronic tool proved to be an effective way to support the involvement of parents in the care planning of their children with ID [46]. In this assumption, the individualization and singularity of each family should be considered in the implementation of family-centered health policies [46,47,55,53,74].

The findings of this study indicate that the involvement of all stakeholders in the effective care of children with ID is relevant to the FCC implementation. From this perspective, the development and benefits achieved by children depend on the family functionality and engagement with health professionals and health services. Presently, the family history is being used to help professionals make treatment decisions, but it also helps family members as they try to navigate the complexity of healthcare systems [73].

The families under study experience increased rates of stress [47,51,50], due to the complexity of daily care to children with ID. A study with parents and their disabled child at home evidence there is a lot to learn and many of the medical procedures that parents carry out are emotionally demanding [75]. Similarly, other studies describe the tension that some families feel between their role as nurse and as a parent [42,76]. Thus, parents are trained through participation and collaboration, which enables them to make responsible and informed decisions [71].

Honoring the diversity of families is also focus of attention by health professionals, described as cultural sensitivity, competency, responsiveness, and humility [77]. The failure to honor the diversity of families can negatively affect assessment and intervention [58]. That's why the focus must be addressed to the family as a unit and not only to the member who needs specific care [60,70,78,79].

To this end, the FCC must improve the partnership approach to decision-making in healthcare [28,34,35], which was also recognized in this study. Significantly, one of the basic principles of FCC is the assumption that the processes of care provision are as important to the success of the child and family outcomes as the specific characteristics of the clinical interventions carried out [79].

However, ambiguity remains on what specific interventions constitute FCC, on both the level of provider and parents [34], and what each participant values most. Parents value the information and tangible/emotional support given to the family by health professionals who have developed trusting relationships with the child [47,[50], [53], [54]]. It is also unclear whether the approach to parents/families involves equally fathers and mothers in the child caregiving. Despite the increasing involvement of fathers in caregiving, as well as its important and positive contribution to child development, research in the field of children with ID is still primarily focused on mothers [[80], [81], [82]]. For instance, while the applications of FCC involving parents are on the rise, these are mainly directed toward and largely used by mothers [51,83], as well as the difficulty in recruiting fathers for studies [50,53,84]. Nevertheless, fathers are presumably still influenced by the illness of their child and may face stress when parenting a chronically ill child [82]. In the study by Lucyshyn et al. [51], mothers and fathers followed programs based on their perspectives, needs and preferences but mothers showed greater outcomes than fathers, with a significant increased perception of family quality of life and decreased parental stress. Especially due to more effective and monitoring daily care. Therefore, it is suggested that FCC approach should optimize efforts to reach the father [50,53] assigning him a key role in care, according to the cultural specificities of each population and the role that fathers plays in society [77]. This involvement can be translated into family satisfaction [47], effecting psychological and social benefits [50,53]. The development of policies can also improve these family's health outcomes, not only in terms of physical accessibility, but also through public awareness of the importance and effective participation of fathers in the healthcare of children with ID [74].

Furthermore, parents of children with more severe deficits are more likely to use healthcare services [5,85], and face more financial burden than parents with healthy children [49]. To address this issue, policies should identify the factors that motivate or prevent them from using services [55,50,74], recognizing that they must be adapted to their needs, characteristics, and interests [52,55,50]. Strong social support has been associated with lower morbidity and mortality, and can be an important area of focus for professionals working with these families that experience less economic and family stability [73]. The evaluation of sociodemographic factors is also fundamental to achieve this goal [55] since parents with lower educational level and economic income benefit from the FCC [50]. Conversely, for Shevell et al. [55], the improvement of family-centered health services can be achieved mainly in the field of education, and in response to the expectations of high socio-economic families. Wakimizu et al. [86] reinforces this evidence, stating that the level of education and household income are important factors associated with family empowerment among families raising a child with special needs. The preferred environment should be on home or community [38], where families ensure their daily lives. Especially through home visit services, families are allowed to control their lives independently, based on their acquired knowledge and long-lasting relationships with other families, friends and health professionals [86]. In any case, whether in the home or hospital environment, FCC provides positive outcomes in families’ well-being [37,87].

This review can guide the practice of FCC care for nurses or other health professionals in any context of pediatric healthcare for families with children with ID. However, these findings suggest that most of the components and health outcomes of the FCC approach in families with children with ID are not exclusive of this population, making them applicable to a variety of conditions (diverse health experiences) and/or other populations [37,48,60].

From a theoretical perspective, this study adds knowledge to the way FCC is conceptualized in these families. Nevertheless, more research is needed to enhance the impact of the FCC approach across specific population/groups, conditions, and care settings [37].

## 5 Limitations

Although our research was conducted in reliable electronic databases, studies in other databases may have been neglected. Also, the full use of studies in English (the only alternative to the Portuguese language), were considered limitations. In this context, the considerable variability in the size of the samples and evaluation method of the FCC approach may have led to some deviations in the elaboration of the results. In the studies, it is also unclear which members constitute the family and which are caregivers. Therefore, the results cannot be generalized to all families.

When excluding articles that raised doubts, the authors were not contacted. Without a more detailed description of the quality of the article, this limitation may have led to the loss of significant articles. Publication bias is one of the limitations of this study, since the trend on this subject in the available scientific publications is more likely to be based on positive evidence than negative, making the results available for comparison biased.

## 6 Conclusion

This paper used an established systematic review methodology to understand the impact of the FCC approach, through its components and health outcomes in families with children with ID. At current, the FCC model is widely used in the pediatric context, but its approach is generic in most of the studies. Through this review, focused specifically on families with children with ID, it was possible to understand which components, inherent to the FCC approach, are particular and relevant on these families. It was emphasized the involvement of these families and the partnership with health professionals, in the effective provision of care for their children with ID. From this viewpoint, the development and benefits achieved by children (as individuals) and the family (as a unit) depend on the functionality of the family and the recognition of their individual needs, characteristics, and interests. Therefore, the focus should be on the family as a unit and not just on the children with ID. A critical change is also needed from the traditional focus on mothers and children to the wider context of the family. This switch is imperative because almost all individuals are attached to families in which each member is connected to and influenced by the others. Only on this assumption, the FCC approach can support families with children with ID. Through effective participation and collaboration, families can make responsible and informed decisions regarding the care provided to their children. Benefits are reported in the well-being and quality of life of the family and, therefore, of the child with ID who is part of it, as well as satisfaction with health services. Nevertheless, more studies are needed to confirm the health outcomes of the FCC approach in these families, with accurate measuring instruments. Similarly, further studies are required to verify the frequency and duration of FCC-related interventions necessary to achieve benefits in the families cared for as a unit.

## Funding

This work is funded by national funds through the Foundation for Science and Technology, under the project UIDP/04923/2020.

## Data availability

No data associated has been deposited into a publicly available repository. Data included in article/supplementary material/referenced in article.

## CRediT authorship contribution statement

**Teresa Dionísio Mestre:** Writing – review & editing, Writing – original draft, Methodology, Investigation, Conceptualization. **Manuel José Lopes:** Writing – review & editing, Methodology, Conceptualization. **David Matias Mestre:** Writing – review & editing, Writing – original draft, Investigation. **Rogério Ferrinho Ferreira:** Writing – review & editing, Investigation. **Ana Pedro Costa:** Writing – review & editing, Investigation. **Ermelinda Valente Caldeira:** Writing – review & editing, Writing – original draft, Methodology, Investigation, Conceptualization.

## Declaration of competing interest

The authors declare that they have no known competing financial interests or personal relationships that could have appeared to influence the work reported in this paper.
