# Supplementary material for: Impact of family-centered care in families with children with intellectual disability: A systematic review
Source: Heliyon. 2024 Mar 15;10(7):e28241. doi: 10.1016/j.heliyon.2024.e28241 (PMC10981057; doi:10.1016/j.heliyon.2024.e28241)
Supplement: Multimedia component 1 [file mmc1.docx]

**Appendix A**

EBSCOhost Web search strategy on the Databases.

**MEDLINE *with Full Text***

| S5 | S1 AND S2 | Limiters - Full Text; Publication Date: 20180101-20230901 Expanders - Apply equivalent subjects  Search modes - Boolean/Phrase | Interface - EBSCOhost Research Databases  Search Screen - Basic Search  Database - MEDLINE with Full Text | **97** |
| --- | --- | --- | --- | --- |
| S4 | S1 AND S2 | Limiters - Publication Date: 20180101-20230901 Expanders - Apply equivalent subjects  Search modes - Boolean/Phrase | Interface - EBSCOhost Research Databases  Search Screen - Basic Search  Database - MEDLINE with Full Text | 132 |
| S3 | S1 AND S2 | Expanders - Apply equivalent subjects  Search modes - Boolean/Phrase | Interface - EBSCOhost Research Databases  Search Screen - Basic Search  Database - MEDLINE with Full Text | 512 |
| S2 | Family Nursing OR Family Centered Care OR Family-Centered Practice OR Family Centered Approach OR Family Centered Intervention OR Family Centered | Expanders - Apply equivalent subjects  Search modes - Boolean/Phrase | Interface - EBSCOhost Research Databases  Search Screen - Basic Search  Database - MEDLINE with Full Text | 57,501 |
| S1 | Intellectual Disability OR Developmental Disabilities OR Disabled Children | Expanders - Apply equivalent subjects  Search modes - Boolean/Phrase | Interface - EBSCOhost Research Databases  Search Screen - Basic Search  Database - MEDLINE with Full Text | 114,344 |

**CINAHL Plus *with Full Text***

| S5 | S1 AND S2 | Limiters - Full Text; Publication Date: 20180101-20230901 Expanders - Apply equivalent subjects  Search modes - Boolean/Phrase | Interface - EBSCOhost Research Databases  Search Screen - Basic Search  Database - CINAHL Plus with Full Text | **97** |
| --- | --- | --- | --- | --- |
| S4 | S1 AND S2 | Limiters - Publication Date: 20180101-20230901 Expanders - Apply equivalent subjects  Search modes - Boolean/Phrase | Interface - EBSCOhost Research Databases  Search Screen - Basic Search  Database - CINAHL Plus with Full Text | 152 |
| S3 | S1 AND S2 | Expanders - Apply equivalent subjects  Search modes - Boolean/Phrase | Interface - EBSCOhost Research Databases  Search Screen - Basic Search  Database - CINAHL Plus with Full Text | 581 |
| S2 | Family Nursing OR Family Centered Care OR Family-Centered Practice OR Family Centered Approach OR Family Centered Intervention OR Family Centered | Expanders - Apply equivalent subjects  Search modes - Boolean/Phrase | Interface - EBSCOhost Research Databases  Search Screen - Basic Search  Database - CINAHL Plus with Full Text | 34,507 |
| S1 | Intellectual Disability OR Developmental Disabilities OR Disabled Children | Expanders - Apply equivalent subjects  Search modes - Boolean/Phrase | Interface - EBSCOhost Research Databases  Search Screen - Basic Search  Database - CINAHL Plus with Full Text | 52,989 |

***Academic Search Complete***

| S5 | S1 AND S2 | Limiters - Full Text; Publication Date: 20180101-20230901 Expanders - Apply equivalent subjects  Search modes - Boolean/Phrase | Interface - EBSCOhost Research Databases  Search Screen - Basic Search  Database - Academic Search Complete | **164** |
| --- | --- | --- | --- | --- |
| S4 | S1 AND S2 | Limiters - Publication Date: 20180101-20230901 Expanders - Apply equivalent subjects  Search modes - Boolean/Phrase | Interface - EBSCOhost Research Databases  Search Screen - Basic Search  Database - Academic Search Complete | 192 |
| S3 | S1 AND S2 | Expanders - Apply equivalent subjects  Search modes - Boolean/Phrase | Interface - EBSCOhost Research Databases  Search Screen - Basic Search  Database - Academic Search Complete | 521 |
| S2 | Family Nursing OR Family Centered Care OR Family-Centered Practice OR Family Centered Approach OR Family Centered Intervention OR Family Centered | Expanders - Apply equivalent subjects  Search modes - Boolean/Phrase | Interface - EBSCOhost Research Databases  Search Screen - Basic Search  Database - Academic Search Complete | 24,767 |
| S1 | Intellectual Disability OR Developmental Disabilities OR Disabled Children | Expanders - Apply equivalent subjects  Search modes - Boolean/Phrase | Interface - EBSCOhost Research Databases  Search Screen - Basic Search  Database - Academic Search Complete | 92,637 |

***Psychology and Behavioral Sciences Collection***

| S5 | S1 AND S2 | Limiters - Full Text; Publication Date: 20180101-20230901 Expanders - Apply equivalent subjects  Search modes - Boolean/Phrase | Interface - EBSCOhost Research Databases  Search Screen - Basic Search  Database - Psychology and Behavioral Sciences Collection | **210** |
| --- | --- | --- | --- | --- |
| S4 | S1 AND S2 | Limiters - Publication Date: 20180101-20230901 Expanders - Apply equivalent subjects  Search modes - Boolean/Phrase | Interface - EBSCOhost Research Databases  Search Screen - Basic Search  Database - Psychology and Behavioral Sciences Collection | 250 |
| S3 | S1 AND S2 | Expanders - Apply equivalent subjects  Search modes - Boolean/Phrase | Interface - EBSCOhost Research Databases  Search Screen - Basic Search  Database - Psychology and Behavioral Sciences Collection | 682 |
| S2 | Family Nursing OR Family Centered Care OR Family-Centered Practice OR Family Centered Approach OR Family Centered Intervention OR Family Centered | Expanders - Apply equivalent subjects  Search modes - Boolean/Phrase | Interface - EBSCOhost Research Databases  Search Screen - Basic Search  Database - Psychology and Behavioral Sciences Collection | 28,351 |
| S1 | Intellectual Disability OR Developmental Disabilities OR Disabled Children | Expanders - Apply equivalent subjects  Search modes - Boolean/Phrase | Interface - EBSCOhost Research Databases  Search Screen - Basic Search  Database - Psychology and Behavioral Sciences Collection | 118,222 |
